# Genetic Predisposition to Coronavirus Disease 2019 in Relation to Ten Cardiovascular Conditions: A Two-Sample Mendelian Randomization Study

**DOI:** 10.3389/fmed.2022.796165

**Published:** 2022-02-17

**Authors:** Min Jia, He-Jia Chen, Ling-Mei Jia, Ya-Li Chen

**Affiliations:** Cardiovascular Medicine Department, The Second Hospital of Hebei Medical University, Shijiazhuang, China

**Keywords:** COVID-19, cardiovascular disease, Mendelian randomization, two-sample, gene prediction

## Abstract

**Background:**

The long-term health consequences of coronavirus disease 2019 (COVID-19) remain largely unclear. This study aimed to apply the Mendelian randomization (MR) design to estimate the causal associations between COVID-19 and ten cardiovascular conditions.

**Methods:**

Single-nucleotide polymorphisms (SNPs) associated with COVID-19 were used as instrumental variables to estimate the causal effect of COVID-19 on ten cardiovascular conditions. The random-effects inverse-variance weighted (IVW) method was conducted for the main analyses with a complementary analysis of the weighted median and MR-Egger approaches.

**Results:**

In the IVW analysis, genetically predicted COVID-19 was suggestively associated with major coronary heart disease events (OR 1.081; 95% CI 1.007–1.16; *P* = 0.045) and heart failure (OR 1.049; 95% CI 1.001–1.1; *P* = 0.045) with similar estimates in weighted median regressions. No directional pleiotropic effects were observed in both funnel plots and MR-Egger intercepts.

**Conclusions:**

Our findings provide direct evidence that patients infected with COVID-19 are causally associated with increased risk of cardiovascular disease, especially for major coronary heart disease events and heart failure.

## Introduction

The outbreak of the coronavirus disease 2019 (COVID-19), which is caused by severe acute respiratory syndrome coronavirus 2 (SARS-CoV-2), is rapidly evolving as a worldwide health crisis. As of October 3, 2021, this worldwide health crisis has directly resulted in more than 212 million confirmed cases with a mortality of 2.3%, which means more than 4.8 million people directly died of COVID-19. Up to now, a lot of studies have revealed a significant observational association between cardiovascular diseases and COVID-19 (1). More importantly, COVID-19 complicated by cardiovascular diseases is reported to associate with a higher risk of adverse outcomes, even mortality (2). However, all these findings are based on observational studies and several limitations existed. First, some confounding factors may affect the reliability of these results, including unmeasured risk factors or other potential uncertainties. Besides, it is not long since the discovery of COVID-19, the long-term effect of COVID-19 on cardiovascular diseases may not be reflected in the previous studies. Therefore, the causal association between COVID-19 and cardiovascular diseases is unclear. However, these pieces of evidence are necessary and important because they can reflect the subsequent social burden and contribute to the government policy on public health.

Mendelian randomization (MR) is a recently emerged technique and conceptually similar to prospective randomized controlled trials, which can be used to assess the causality between the risk factor and particular disease (3–5) due to its advantages in avoiding the potential bias (6). In the present study, we assessed whether COVID-19 is causally associated with increased risk of ten cardiovascular conditions using a two-sample summary MR.

## Methods

### Overall Study Design

**Figure 1:**
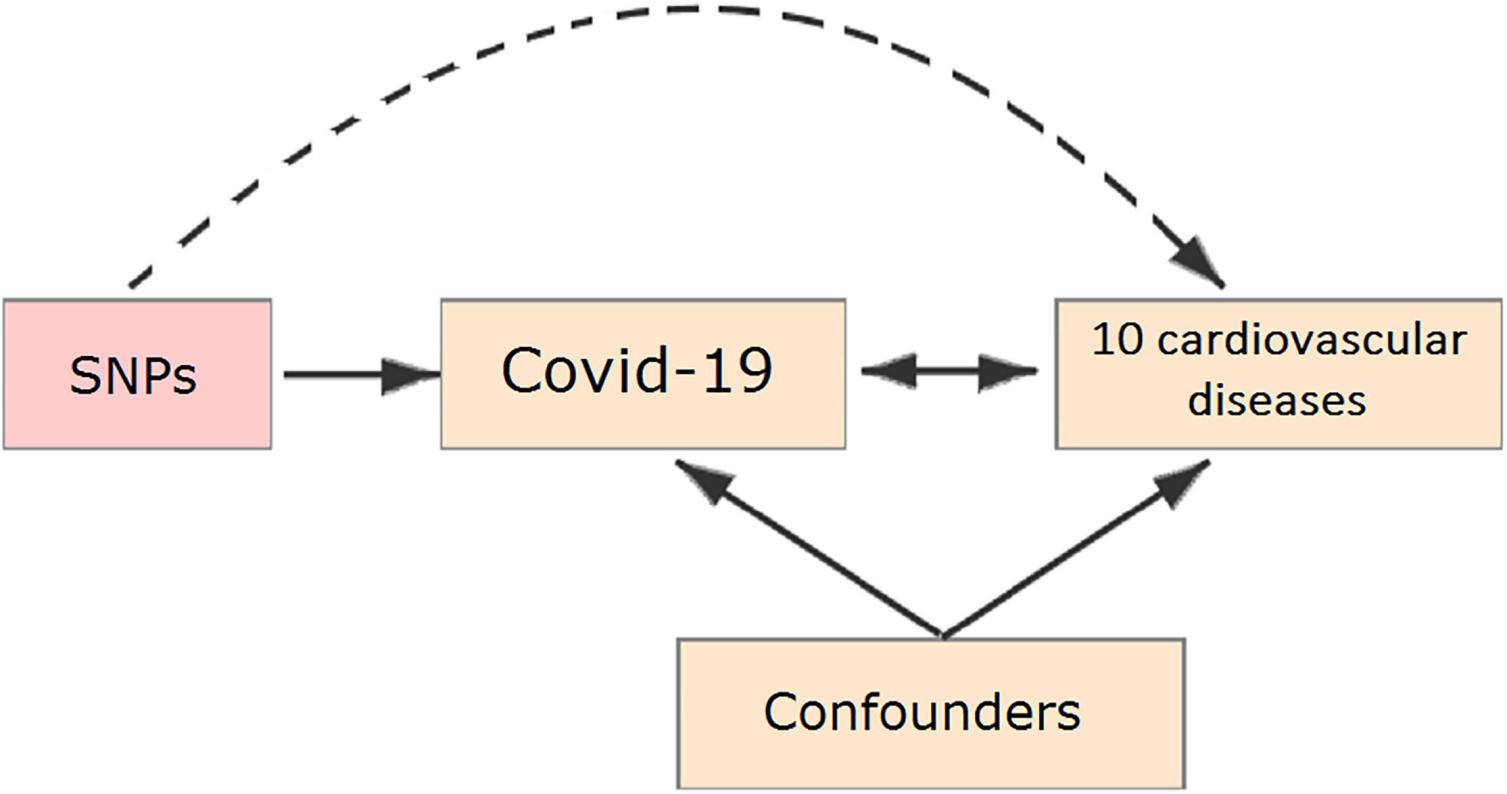
Schematic representation of a Mendelian randomization (MR) analysis. We selected single-nucleotide polymorphisms (SNPs) associated with coronavirus disease 2019 (COVID-19) and the corresponding effect for these SNPs was estimated based on the risk of 10 cardiovascular diseases.

In our study, all the summary data was obtained from publicly published studies. Their institutional review committee has approved their design and data in respective studies. Therefore, no further sanction was required in the present study. Two-sample MR (7–9) was used to assess the causal effect of COVID-19 on the risk of ten cardiovascular diseases, the schematic view of the study design for two-sample MR analyses in this study is shown in Figure 1.

### Data Sources

#### Identification of SNPs Associated With COVID-19

Summary-level genetic data for COVID-19 were acquired from results of the Genetics of Mortality in Critical Care (GenOMICC) genome-wide association study (GWAS) (10), which included 2,244 critically ill COVID-19 patients from 208 UK intensive care units (ICUs) and 11,220 random controls matched by ancestry from UK Biobank. In total, eight single-nucleotide polymorphisms (SNPs) associated with COVID-19 were obtained as instrumental variables (Supplementary Table 1), identified from the primary meta-analysis of 13,464 individuals based on the genome-wide significant level (*P* < 5 × 10^−8^) (11). To identify relatively more independent genome-wide significant SNPs, we excluded SNPs in linkage disequilibrium with the other SNPs (*r*^2^ < 0.005), and only SNPs in both the exposure and outcome GWAS datasets were included in our analysis.

#### Study Outcome: Cardiovascular Disease

Corresponding data for cardiovascular diseases were obtained from the FinnGen project (FinnGen, Finland), which was used to extract the summary data set for GWAS of cardiovascular diseases. FinnGen study launched in Finland in the autumn of 2017 is a unique study that combines genome information with digital health care data. The FinnGen study is an unprecedented global research project representing one of the largest studies of this type. This data freeze consists of 176,899 individuals, almost 17,000,000 variants and 2,444 disease endpoints (https://www.finngen.fi/en/access_results). To be able to determine the differential cardiovascular risk associated with COVID-19, we analyzed a broad range of cardiovascular diseases, including aortic aneurysm, thrombo-embolic diseases (deep vein thrombosis and pulmonary embolism), and other cardiovascular diseases (major coronary heart disease event, atrial fibrillation, heart failure, peripheral artery disease, primary hypertensive diseases, rheumatic valve diseases, and non-rheumatic valve diseases). Due to no individual patient data being available, we used summary data for cardiovascular diseases.

### Statistical Analysis

Since there is no individual-level GWAS data available, two-sample MR analyses were used to assess the causal association between COVID-19 and 10 cardiovascular diseases based on the summary-level genetic data.

In the principal analyses, an inverse-variance weighted meta-analysis with a random-effects model was used (12). As a first sensitivity analysis, potential outlier SNPs (*P* < 0.1) were excluded, identified by MR Pleiotropy RESidual Sum and Outlier (MR-PRESSO) method (13). In a second sensitivity analysis, both weighted median (14) and MR-Egger methods (15) were used to ensure lower pleiotropy in the present study. Two-tailed was used in all statistical tests. To account for multiple testing in our primary analyses of COVID-19 in relation to the 10 outcomes, a Bonferroni-corrected threshold of *P* < 5 × 10^−3^ (a = 0.05/10 outcomes) was used in our analysis. Associations with *P*-values between 5 × 10^−3^ and 0.05 were considered suggestive evidence of associations, which required further confirmation.

To probe the total effect of COVID-19 on cardiovascular disease and cerebrovascular disease, a meta-analysis was made. All exposure-specific MR analyses were performed for each cardiovascular disease from the FinnGen project and then were meta-analyzed to generate the pooled estimates for COVID-19 on the risk of cardiovascular disease and cerebrovascular disease, separately. The *I*^2^ statistics and corresponding *p*-value derived from Cochran's Q test were used to quantify heterogeneity between estimates from different diseases. Random-effect model meta-analyses were used in our study to pool instrumental variable estimates in the effect of COVID-19 on different diseases. R-based “meta” package was used in all of our meta-analyses.

Based on a reasonable request, the related data and statistical coding can be obtained from the corresponding author. The MR software packages TwosampleMR (0.5.6) and R version 4.0.3 (2020-10-10) (Vienna, Austria) were used in our study (13, 16).

## Results

### Participants and Genetic Instrumental Variables for COVID-19

The mean age of the 2,244 patients with COVID-19 included in the present analysis was 57.3 years and 69.74% were men. As shown in Supplementary Table 1, we presented all genetic instruments associated with COVID-19 on the genome-wide significant level (*P* < 5 × 10^−8^). None of the eight SNPs had previously been reported to play a part in any pathway.

### MR Analysis

**Figure 2:**
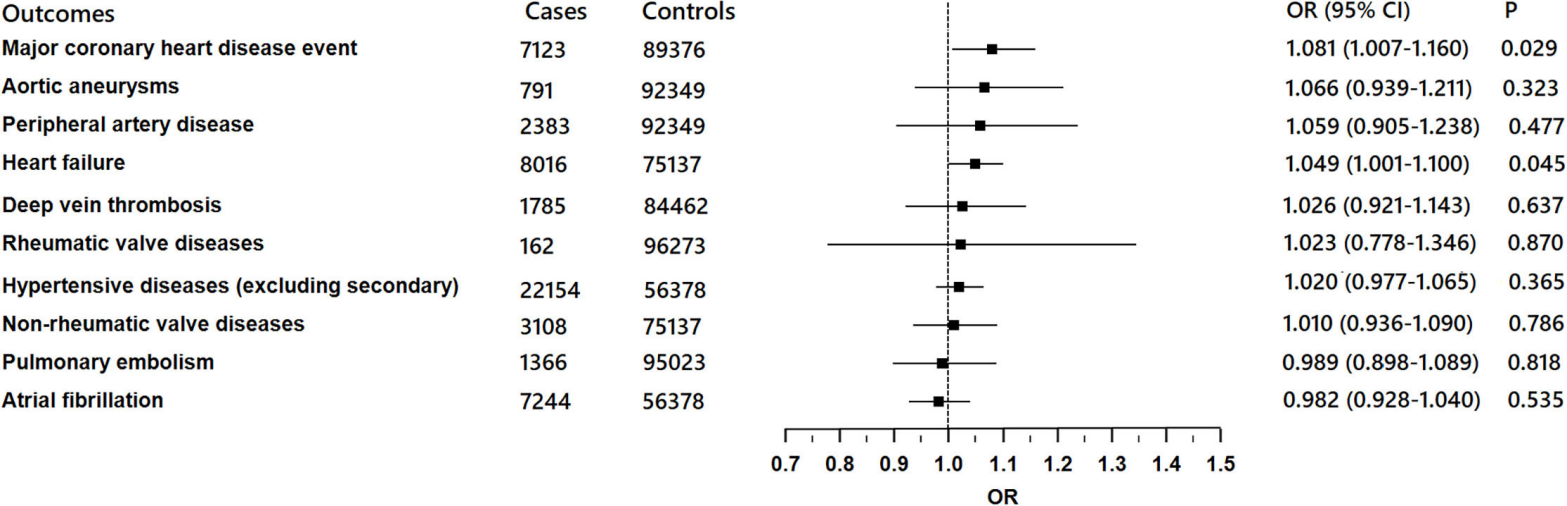
Associations of genetically predicted COVID-19 with ten cardiovascular conditions in FinnGen project.

**Figure 3:**
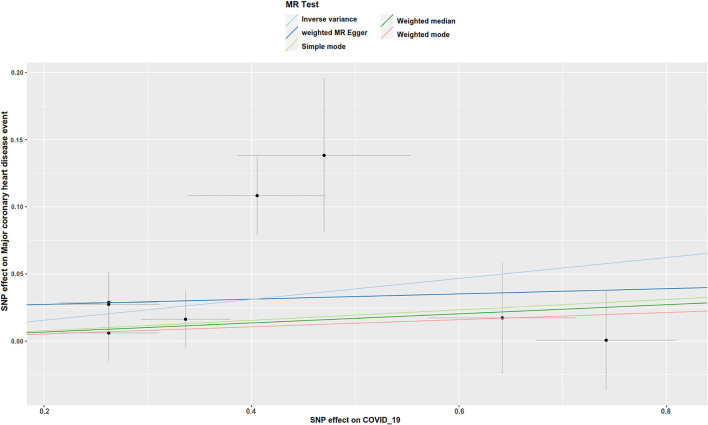
Scatter plot to visualize the causal effect of COVID-19 on the risk of major coronary heart disease events. The slope of the straight line indicates the magnitude of the causal association. IVW, inverse-variance weighted.

**Figure 4:**
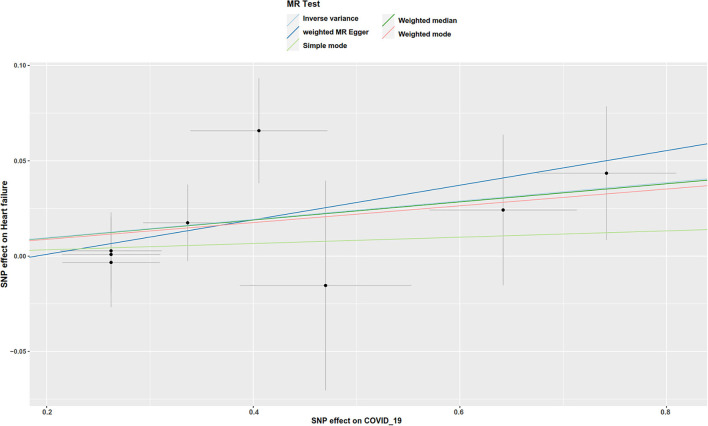
Scatter plot to visualize the causal effect of COVID-19 on the risk of heart failure. The slope of the straight line indicates the magnitude of the causal association.

There was suggestive evidence of a positive association between genetically predicted COVID-19 and major coronary heart disease events (OR 1.081; 95% CI 1.007–1.16; *P* = 0.029; Figures 2, 3), heart failure (OR 1.049; 95% CI 1.001–1.1; *P* = 0.045; Figures 2, 4), separately. Whereas, no association was observed between COVID-19 and aortic aneurysms, peripheral artery disease, deep vein thrombosis, pulmonary embolism, rheumatic valve diseases, non-rheumatic valve diseases, and atrial fibrillation (Figure 2). The MR-PRESSO method identified one outlier SNP for major coronary heart disease events and two outlier SNPs for peripheral artery disease. Outlier correction did not materially change the OR estimates for major coronary heart disease events (1.053; 95% CI 0.998–1.11) or peripheral artery disease (1.11; 95% CI 0.958–1.286). There are no outlier SNPs identified using MR-PRESSO to analyze the other outcomes. The OR estimates of the weighted median analysis (Supplementary Table 2) were similar to those of the standard MR analysis (inverse variance weighted method) but of low precision. The MR-Egger analysis for most outcomes revealed consistent estimates but with lower precision, and without indication of directional pleiotropy (Supplementary Table 2).

## Discussion

In this present study, we assessed the causal effect of COVID-19 on a wide range of cardiovascular conditions and found evidence that COVID-19 is causally associated with an increased risk of major coronary heart disease events and heart failure.

Coronavirus disease 2019 (COVID-19) is characterized by a long incubation period, high transmission, and diverse clinical manifestations (17), which has rapidly evolved as a major threat to global health and has affected the lives of billions of individuals since it was first reported in December 2019. In addition to infecting the respiratory system, lots of studies have also revealed an observational association between COVID-19 and cardiovascular disease (1). Not only acute myocardial injuries (18) but also chronic damage to the cardiovascular system (19) may be caused by COVID-19 (17). However, all of these findings are based on observational studies and some confounding factors may potentially cause a limitation to generalizing these findings.

MR is a recently emerged technique, which is conceptually similar to prospective randomized controlled trials (RCT) because all the inherited genetic variants are determined prior to the disease onset. Our present MR analysis can provide a good solution to avoid potential confounding factors and assess the causal effect of COVID-19 on cardiovascular disease. Based on summary statistics from the newest GWAS studies for COVID-19 (*n* = 13,464) and cardiovascular conditions (up to *n* = 176,899 individuals), there is a causal effect of COVID-19 on the risk of major coronary heart disease event and heart failure, conferring 8.1 and 4.9% increased risk, respectively.

Despite a low level of scientific evidence on this subject, many studies had revealed an association between coronary heart disease and the infection by SARS-CoV-2 (20, 21). There are some hypotheses raised so far to clarify the relationship between infection of COVID-19 and the increased risk of ischemic events (22). Angiotensin-converting enzyme 2 (ACE2), which acts as a receptor for the virus, is mostly present in the lungs but also present in great amounts in the heart, resulting in cardiovascular (CV) complications (23). Besides, the systemic inflammation promoted by SARS-CoV-2 may further lead to a high risk of myocardial and vascular injury with an increase of N-terminal prohormone of brain natriuretic peptide (NT-proBNP) and troponin, and consequently CV complications (22). The combination of exacerbated inflammation and other factors, including immobilization, hypoxemia, and in some cases DIC can eventually culminate in a prothrombotic state (24), which may play an important part in the occurrence and development of coronary heart disease (25, 26). In addition, a last adverse mechanism may have existed in the clinical practice. Patients previously submitted to angioplasty may have a higher risk of recurrent coronary heart disease, such as type-4b acute myocardial injury, due to the hypercoagulability state induced by the infection of SARS-CoV-2 (25, 26).

In the present study, genetically determined COVID-19 is causally associated with increased risk for heart failure, conferring a 67% increased risk. Heart failure has been reported as the major cause of death in patients with COVID-19. Several myocardial aggression mechanisms are involved in the development of heart failure in patients with COVID-19, such as viral direct myocardial injury, O_2_ supply-demand imbalance, indirect and direct inflammatory damage (21, 27). Moreover, the increase of serum troponin was associated with an increase of plasmatic NT-proBNP levels, which further contributed to higher mortality (21, 27).

Notably, our results revealed that there is a lifetime increased risk of cardiovascular disease due to the genetic predisposition of COVID-19 because the genetic variants of one person will not change over a whole lifetime once it occurred. Therefore, the present results may not recapitulate exactly the same as the previous observational effect, but rather provide evidence about the long-term effect of COVID-19 on cardiovascular diseases.

A chief strength of this study is that we assessed the causal associations between COVID-19 and a wide range of cardiovascular diseases in the same study population using the MR method. Another strength is that all of the summary data about cardiovascular diseases were extracted from European ancestry populations. Besides, the genetic variants of COVID-19 were also widely acknowledged by other researchers (5, 10) and more than 74.69% of patients have consisted of European ancestry patients. To further assure the reliability of our analysis, only SNPs that reached genome-wide significance in European ancestry populations were used in our study as recommended (5). Therefore, the potential confounder, which may influence our results, is small in the present study. Pleiotropy is an important limitation of MR analysis, which means a genetic variant may not only contribute to only one phenotype. Fortunately, there is no evidence of directional pleiotropy observed in the present MR. Of course, there are several limitations involved in our study. First, not all cardiovascular diseases were analyzed due to no availability of GWAS data. Besides, the sample size of some outcomes was small. Therefore, weak associations due to insufficient power may have been overlooked in the present study. Most importantly, this study was finished based on summary data of European ancestry populations, whether it is applicable in other ancestry populations needs further verification.

In conclusion, using MR analysis, we found potential evidence about the causal association between the genetic predisposition to COVID-19 and the increased risk of cardiovascular diseases, especially for major coronary heart disease events and heart failure.

## Data Availability Statement

The datasets presented in this study can be found in online repositories. The names of the repository/repositories and accession number(s) can be found in the article/Supplementary Material.

## Ethics Statement

Ethical approval was not provided for this study on human participants because in our study, all the summary data was obtained from publicly published studies. Their institutional review committee has approved their design and data in respective studies. Therefore, no further sanction was required in the present study. The patients/participants provided their written informed consent to participate in this study.

## Author Contributions

Y-LC designed the study and revised the manuscript. MJ finished the analysis. H-JC helped with the use of software. L-MJ, H-JC, and MJ finished the draft of this manuscript. All authors contributed to the article and approved the submitted version.

## Conflict of Interest

The authors declare that the research was conducted in the absence of any commercial or financial relationships that could be construed as a potential conflict of interest.

## Publisher's Note

All claims expressed in this article are solely those of the authors and do not necessarily represent those of their affiliated organizations, or those of the publisher, the editors and the reviewers. Any product that may be evaluated in this article, or claim that may be made by its manufacturer, is not guaranteed or endorsed by the publisher.
