# Supplementary material for: Genetic Predisposition to Coronavirus Disease 2019 in Relation to Ten Cardiovascular Conditions: A Two-Sample Mendelian Randomization Study
Source: Front Med (Lausanne). 2022 Feb 17;9:796165. doi: 10.3389/fmed.2022.796165 (PMC8891370; doi:10.3389/fmed.2022.796165)
Supplement: Supplementary file 1 [file Data_Sheet_1.pdf]

Supplementary Table 1. List of Genetic Instruments for COVID-19 by Each Instrumental SNPs (GWAS Significance with $P < 5\times10^{-8}$ and linkage disequilibrium threshold with $R^2 <$ 0.005)

No.	SNP	Locus	Chr.	EA	OA	EAF	β（SE）
1	rs73064425	LZTFL1	3	T	C	0.07	0.742（0.068）
2	rs9380142	HLA-G	6	A	G	0.69	0.262（0.049）
3	rs143334143	CCHCR1	6	A	G	0.07	0.642（0.071）
4	rs3131294	NOTCH4	6	G	A	0.86	0.405（0.066）
5	rs10735079	OAS1/3	12	A	G	0.63	0.262（0.047）
6	rs2109069	DPP9	19	A	G	0.32	0.336（0.043）
7	rs74956615	TYK2	19	A	T	0.05	0.470（0.083）
8	rs2236757	IFNAR2	21	A	G	0.28	0.262（0.048）

Chr. indicates chromosome; EA, effect allele; OA, other allele; EAF, effect allele frequency.

Supplementary Table 2. Associations between genetically predicted COVID-19 and cardio-cerebrovascular diseases in sensitivity analyses using the weighted median and MR-Egger methods

Outcome	Weighted median		MR-Egger			
	OR (95% CI)	*P* value	OR (95% CI)	*P* value	Intercept[a]	*P* value
Aortic aneurysms	1.061 (0.903-1.248)	0.481	0.998 (0.708-1.406)	0.992	1.027 (0.903-1.169)	0.685
Deep vein thrombosis	1.095 (0.959-1.251)	0.177	1.099 (0.808-1.494)	0.549	0.972 (0.866-1.092)	0.640
Pulmonary embolism	0.958 (0.893-1.028)	0.227	0.897 (0.771-1.043)	0.156	1.038 (0.980-1.098)	0.200
Major coronary heart disease event	1.038 (0.975-1.105)	0.25	1.019 (0.838-1.240)	0.853	1.024 (0.952-1.103)	0.524
Atrial fibrillation	0.980 (0.866-1.109)	0.747	0.927 (0.714-1.203)	0.566	1.026 (0.931-1.132)	0.599
Heart failure	1.049 (0.987-1.115)	0.126	1.094 (0.965-1.240)	0.162	0.983 (0.938-1.030)	0.482
Peripheral artery disease	1.063 (0.952-1.186)	0.272	0.948 (0.607-1.483)	0.817	1.045 (0.883-1.237)	0.604
Hypertensive diseases (excluding secondary)	0.988 (0.877-1.114)	0.254	1.011 (0.966-1.058)	0.633	1.013 (0.968-1.060)	0.575
Non-rheumatic valve diseases	1.067 (0.985-1.156)	0.109	1.124 (0.920-1.373)	0.25	0.958 (0.889-1.032)	0.259
Rheumatic valve diseases	1.022 (0.704-1.483)	0.908	0.712 (0.340-1.490)	0.367	1.158 (0.877-1.530)	0.299

CI, confidence interval; OR odds ratio.

[a]The MR-Egger intercept quantifies the effect of directional pleiotropy. Values that significantly differ from zero provide evidence that the COVID-19-associated single-nucleotide polymorphisms may influence the outcome through other pathways than through COVID-19.
